# Temperature‐Dependent Shifts in Multiple Indirect Defensive Interactions on Black Cherry

**DOI:** 10.1002/ece3.72151

**Published:** 2025-09-18

**Authors:** Emma Dawson‐Glass, Nathan J. Sanders, Marjorie G. Weber

**Affiliations:** ^1^ Department of Ecology and Evolutionary Biology University of Michigan Ann Arbor Michigan USA

**Keywords:** defense mutualisms, multitrophic, plant defense, temperature, tri‐trophic interactions, warming

## Abstract

Many plants engage in indirect defense via tri‐trophic interactions whereby plants provide resources such as food or shelter to mutualists in exchange for protection against herbivores and pathogens, increasing plant fitness. As temperature regimes shift under climate change, understanding the influence of temperature on tri‐trophic defensive interactions is increasingly important. However, where plant species host multiple tri‐trophic defensive interactions, we still lack an understanding of if each interaction, even within the same system, responds in the same way to temperature. In this study, we monitored black cherry (*Prunus serotina)* seedlings for 10 weeks under ambient and increased temperatures to explore the effects of temperature on two different tri‐trophic defensive interactions between black cherry and: (1) mutualistic leaf domatia‐dwelling mites and leaf fungi; and (2) arthropod predators and herbivores. We found that the positive association between mite abundance and domatia size increased by 8.7% on warmed plants, while warming weakened the positive relationship between mite abundance and the abundance of foliar fungi by 14%, though warmer conditions alone did not affect the abundances of any of these groups. Further, warming increased the abundance of arthropod predators by 116% and decreased the amount of herbivory plants experienced by 42%, but did not modify the impact predators had on herbivory. Ultimately, the differences among interacting species with warming did not translate to differences in plant growth, indicating black cherry can be robust to at least some of the variation in species interactions caused by changing temperatures in the short term. These findings illustrate that warming can modify the abundance of, and relationships between, some but not all tri‐trophic defensive interactions in a given system, further confirming that temperature does not impact plant interactions uniformly.

## Graphical abstract

This study examined how temperature mediates multiple tri‐trophic defensive interactions involving black cherry ( *Prunus serotina* ) seedlings. Warmer temperatures altered the strength of some interactions—modifying the relationships between leaf domatia, mutualistic domatia‐dwelling mites, and foliar fungi—while other relationships, like the interactions between predators and herbivores, remained unchanged. Despite these shifts, plant growth was unaffected, suggesting that black cherry may be resilient to some temperature‐driven differences in species interactions.

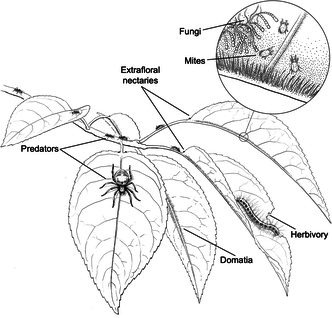

## 1 Introduction

Many plants rely on mutualisms with other organisms for defense against their enemies (Janzen 1966; Bentley 1977; Bronstein 1998, 2015). In many cases, plants provide rewards such as food or shelter to mutualists—typically predators of the plant's enemies—in exchange for protection from herbivores and pathogens (Heil 2008; Quintero et al. 2013; Bronstein 2015; Pearse et al. 2020), forming a tri‐trophic interaction among plants, their enemies, and the consumers of those enemies. Although tri‐trophic defensive interactions can enhance plant fitness, their magnitude and outcomes can depend on biotic and abiotic context (Bronstein 1994; Chamberlain et al. 2014; Maron et al. 2014; Hoeksema and Bruna 2015; Wetzel et al. 2023). Tri‐trophic interactions may be especially variable across contexts because they involve multiple species whose abundances and behaviors can be sensitive to different environmental conditions (Bronstein 1994). Given the ecological importance of tri‐trophic defensive interactions, but also their potential to vary under changing environmental contexts, studies elucidating how these interactions shift under different abiotic conditions are critical in the face of changing climates.

Temperature is one environmental driver central to shaping the behavior (Stuble et al. 2013; Roeder et al. 2022; Hu et al. 2024), physiology (Gillooly et al. 2001; Angilletta Jr. et al. 2004), and phenology of organisms (Thackeray et al. 2016), which can in turn influence the magnitude and direction of tri‐trophic interactions. As climate change alters temperature regimes, warmer conditions have the potential to alter tri‐trophic interactions by modifying the abundances of interacting species. For example, higher temperatures could increase the densities of plant enemies, such as fungal pathogens (Liu et al. 2019; Gallego‐Tévar et al. 2024), modify the attendance of plant defenders, such as mutualistic ants (Fitzpatrick et al. 2014; Tamashiro et al. 2019), or alter plant reward availability, such as extrafloral nectary abundance (Magnoli et al. 2023). These shifts can culminate in disrupting tri‐trophic defense interactions, with potentially negative consequences for plant fitness (Marquis et al. 2014).

Many plants interact with more than one mutualist at a time (Afkhami et al. 2014; Stanton 2003; Keller et al. 2018), and while responses to increased temperatures may vary across species, they may also vary across interactions (Kharouba et al. 2018; Magnoli et al. 2023). In many cases, plants may even host multiple, unique tri‐trophic defensive interactions (Heil 2008), each of which may vary in its responsiveness to temperature. While evidence suggests that warming can alter at least some tri‐trophic defensive interactions (Marquis et al. 2014; Barton and Ives 2014; Magnoli et al. 2023), where plant species host multiple tri‐trophic defensive interactions, it is unclear if each interaction will respond similarly to warming. As such, exploring the responses of multiple tri‐trophic defensive interactions within the same system would improve our understanding of which trophic groups are most responsive to elevated temperatures and the impact these responses have on indirect defense.

**FIGURE 1:**
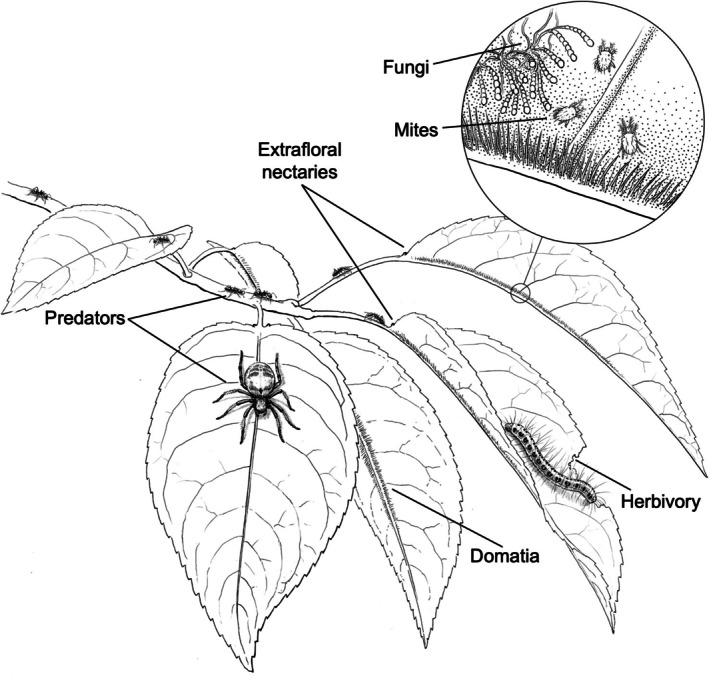
An illustration of the key plant traits and organisms involved in the two focal interactions with black cherry. Illustration by John Megahan.

Black cherry ( *Prunus serotina* ) hosts multiple tri‐trophic defensive interactions that could be impacted by shifts in temperature (Figure 1), and as such, is a powerful study system to test the effect of warming across multiple interactions while holding the system constant. In one mutualism, foliar mites reside in domatia on the undersides of black cherry leaves (Figure 1), which reduces abiotic stress for the mites (Grostal and O'Dowd 1994; Onzo et al. 2010). The domatia‐dwelling mites defend their host plant by consuming pathogenic fungi and small herbivores (Norton et al. 2000; Romero and Benson 2005). Mites are generally sensitive to abiotic conditions, particularly those that increase desiccation risk (Grostal and O'Dowd 1994; Ghazy and Suzuki 2014). Thus, warming could reduce mite abundance on leaves. Conversely, warming may make conditions more favorable for some foliar fungi (Liu et al. 2019; Gallego‐Tévar et al. 2024), potentially increasing the abundance of fungi on leaves.

In a second category of mutualistic interactions, black cherry supports a suite of arthropod predators, including several different ant and spider species, which contribute to the regulation of herbivore populations (Way and Khoo 1992; Romero et al. 2008). Extrafloral nectaries—small sugar‐secreting glands on leaves of black cherry—are available as food rewards to generalist predators, which in turn can protect black cherry from herbivores (Tilman 1978). However, whether these interactions between predators, herbivores, and plants depend on temperature has not been studied in black cherry. In other systems, the abundance of arthropod predators generally increases with warming (Prather et al. 2018; Høye et al. 2020; Magnoli et al. 2023; Hu et al. 2024), as well as the top‐down impacts of predators (Barton et al. 2009; Romero et al. 2018; Walker et al. 2020). In one annual forb, warming also altered the abundance of extrafloral nectar, increasing the abundance of predators attracted to the nectar (Magnoli et al. 2023).

Here, we capitalize on two tri‐trophic defensive interactions with black cherry to evaluate how multiple tri‐trophic interactions respond to increases in temperature using a field‐based simulated warming experiment, assessing differences in black cherry, its arthropod mutualists, and its pests and pathogens. We examine whether different tri‐trophic defensive interactions respond in parallel to shifts in abiotic conditions, or if these interactions respond differently, despite their functional similarity and shared host. Specifically, we investigated two interrelated questions:

1. What are the direct effects of warming on the interacting species and their relevant traits? Specifically, we asked whether warming impacts the abundance of foliar mites, fungi, herbivory, and predators on leaves, as well as investment in domatia, extrafloral nectaries, and plant growth.
2. Does warming alter the relationships among multitrophic interactors? Specifically, we asked whether warming modifies the relationships among (a) domatia, mites, and fungi and (b) extrafloral nectaries, arthropod predators, and herbivory.

## 2 Methods

### 2.1 Study System

Black cherry ( *Prunus serotina* , family Rosaceae) is a deciduous tree species with a native range that includes the eastern United States and parts of the southwestern United States and Mexico, and is common in both forests and open areas (Voss 1985). In addition to cyanogenic tissue that aids in direct defense against enemies (Voss 1985), black cherry also possesses traits that aid in mediating indirect defense by arthropod mutualists. The first, leaf domatia, presents as a strip of pubescence along both sides of the midrib on the leaf abaxial surface (Figure 1). The second, extrafloral nectaries, present as glands on the petiole, below the base of the lamina (Chin et al. 2013; Figure 1). These traits mediate interactions between mutualistic mites, including species in the Phytoseiidae family (Garon‐Labrecque 2017), and ants, including species in *Formica, Prenolepis*, and other genera (Dawson‐Glass, personal observation; Tilman 1978). Spiders also occur on black cherry (Larcenaire et al. 2021). A wide variety of herbivores feed on black cherry, including *Malacosoma americanum* (Tilman 1978; Abarca and Lill 2015), *Hyphantria cunea* (Fitzgerald 2008), and *Hydria prunivorutu* (Marquis 1990). Black cherry also hosts fungi on its leaves, including pathogens such as powdery mildew (Santiago‐Santiago et al. 2014) and black cherry leaf spot (Marquis 1990).

### 2.2 Experimental Design

We planted 96 black cherry seedlings into the Long‐Term Ecological Research warming arrays at the Kellogg Biological Station (42.4, −85.4) in southwest Michigan, USA. Seedlings were obtained from Cold Stream Farms (Free Soil, MI) and were sourced from natural populations. The mean annual temperature is 9.7°C, and the annual precipitation of 890 mm per year. The specific site where the experiment took place is an old‐field plant community, which was mowed and cleared before the experiment was initiated.

The site comprised eight experimental arrays, each consisting of a 3‐m circular plot. Four of the experimental arrays were actively warmed by infrared heaters, and four arrays were unwarmed controls with structures added to control for the effects of shading by the heaters. In experimentally warmed arrays, temperature was artificially elevated using six ceramic infrared heaters per plot (Kalglo Inc. Model FTE‐1000), which used a proportional‐integrative‐derivative control system (Kimball et al. 2008; Zettlemoyer et al. 2019; Magnoli et al. 2023). Temperatures were elevated 2°C–3°C above ambient conditions in line with current climate warming projections by the end of the century for the region (IPCC 2023). Average daytime and nighttime temperatures in control plots during our study were 23.7°C and 16.2°C, respectively.

In June 2018, 12 seedlings were planted in each array, such that there was a total of 48 plants that received the warming treatment and 48 plants that received the control treatment. At the time of planting, we measured initial seedling height. The seedlings were in the field for 10 weeks. Starting in the second week of the experiment, we counted the number of arthropod predators (ants and spiders), the total number of leaves, and the number of leaves showing evidence of herbivory once per week for each seedling. We did not collect data in week eight due to heavy precipitation impacting field safety at the site. We measured final seedling height at the end of the growing season (week 10, August 2018).

To quantify the number of extrafloral nectaries, domatia size, the number of mites, and the abundance of fungi (Figure 1), at the end of the 10‐week growing period, we collected three leaves from each seedling, resulting in data from 250 leaves total. Each leaf was immediately placed into individual plastic bags with a moist paper towel (to prevent leaves and organisms that occur on the leaf surface from desiccating) and kept on ice until being transferred to a 4°C refrigerator before being processed. Within 24 h of collection, the mite and fungus communities were assessed. Mites were counted by examining the abaxial surface of each leaf using a dissecting microscope and recording the number of mites observed. We quantified fungal growth on the leaf surface using a fungal peel method (Harris 2000; Monks et al. 2007). Specifically, a 19 mm wide piece of Scotch Tape (Scotch Brand, Hutchinson, Minnesota, USA) was pressed on the abaxial leaf surface and then peeled off to remove mycelia. The tape was then attached to a microscope slide and stained using a 0.5% (w/v) trypan blue solution in lactoglycerol (1:1:1 lactic acid, glycerol, and water filtered at 0.45 μm) (Graham et al. 2022). Under a compound microscope, we then counted the number of hyphal strands that crossed a transect of the tape along the 19 mm width of the tape. We then measured domatia size and extrafloral nectary abundance for each leaf. We assessed if there were domatia present on each leaf and measured domatium length in millimeters under a dissecting microscope using a micrometer if domatia were present. We measured domatia size as the total length of the midrib with pubescence on either or both sides of the midrib. We then counted the total number of extrafloral nectaries along the petiole on each leaf.

### 2.3 Statistical Analyses

For all analyses, we included only data from plants that were still alive at the end of the experiment, resulting in data from 83 seedlings total. For data on mite abundance, fungal abundance, domatia size, and extrafloral nectary abundance, which we collected from a subset of three leaves on each plant, we aggregated data to a single value per plant. For mite abundance, fungal abundance, and extrafloral nectary abundance, we summed the data from the three leaf samples to get a single abundance value per plant. We summed values rather than using an average to ensure count data were represented as integers and could be modeled using a Poisson distribution, as is standard for count data (described further below in *How does warming impact interactions?*). For the domatia size, we took an average value from the three leaves to get a single value per plant. We accounted for the experimental design of our study by incorporating plot as a random effect in all analyses. All analyses were performed in R version 4.3.1 (R Core Team 2023).

### 2.4 What Are the Direct Effects of Warming?

To assess the direct effects of warming on each trophic group involved in black cherry's tri‐trophic defensive interactions, we calculated log response ratios for each species and trait measured. Specifically, for both the warmed and control treatments separately, we calculated the mean values of domatia length, mite abundance, fungal abundance, total extrafloral nectaries on collected leaf samples, total predator abundance over the 10‐week experiment, proportion of leaves with herbivory in week 10 of the experiment, and seedling relative growth rate. We then calculated the log response ratio of each variable as:

$$\ln\left(\frac{\text{Mean in warmed plots}}{\text{Mean in control plots}}\right)$$

We calculated the standard error of the log response ratio following Hedges et al. (1999). We then multiplied the standard error value by the critical value for 95% confidence intervals to calculate confidence intervals for each effect size (Hedges et al. 1999). To assess if a trophic group differed in abundance or size between treatments, we assessed if the confidence intervals overlapped zero, and we considered an effect size significant if its confidence intervals did not overlap zero.

We also quantified the effect of warming on plant mortality using a generalized linear mixed effects model with a binomial distribution. We modeled survival as a binary response variable and used treatment as a fixed effect and plot as a random effect.

### 2.5 Does Warming Alter the Relationships Among Multitrophic Interactors?

To assess the effects of warming on multitrophic interactions, and whether these interactions influence plant growth, we used mixed effects models. In particular, we created separate models for each defensive mutualist (i.e., arthropod predators and mites), plant enemy (i.e., herbivore damage and fungal abundance), and plant growth, to explore how associations between trophic groups were modified by warming. For models with the proportion of leaves with herbivory and plant growth as response variables, we used linear mixed effects models (lme, nlme package; Pinheiro et al. 2025; Table 1). For models with mite abundance, fungal hyphae abundance, and predator abundance as response variables, we used generalized linear mixed effects models (glmer function, lme4 package; Bates et al. 2025) with a Poisson distribution (Table 1). Predictor variables in all models included warming treatment, the species or trait(s) we expected to directly influence the abundance of the response variable, and their interaction (Table 1). Where the interaction term was non‐significant, we removed it from the model. For plant growth and herbivory, we used a square root transformation to normalize skewed distributions. While we note that, in addition to fungivory, mites can act as predators to herbivores (Grostal and O'Dowd 1994; Agrawal and Karban 1997; Romero and Benson 2005), we did not detect a significant effect of mite abundance on herbivory when we included it in our model of herbivory (*t* = 0.01, *p* = 0.99), and including it as a predictor resulted in a higher AIC score (AIC including mites = 36.9, AIC without mites = 27.8). Therefore, we opted not to include mite abundance in our model of herbivory.

**TABLE 1:** Description of the models used to examine the effects of warming, the species or trait(s) we expected to directly influence the abundance of the response variable, and their interaction on each trophic group, including details of the error distributions and fixed effects used for each response variable. Note that all models included plot as a random effect, the mite, fungal, and predator abundance models included leaf replicates as a random effect, and all variables modeled with a Gaussian distribution were square root transformed.

Response variable	Error distribution	Fixed effects
Mite abundance	Poisson	Treatment × Domatia size
Fungal abundance	Poisson	Treatment × Mite abundance
Predator abundance	Poisson	Treatment × Extrafloral nectary abundance
Herbivore damage	Gaussian	Treatment × Predator presence
Plant growth	Gaussian	Treatment × Herbivore damage + Treatment × Fungal abundance

We included plot (i.e., experimental array number) as a random effect for all models. In six instances, there were only two leaf samples available per plant, rather than the standard three we collected for the rest of the plants. This was because these seedlings did not have more than two leaves at the time of collection. In these instances, leaf‐level data (mite abundance, fungal abundance, domatia size, extrafloral nectary abundance) were aggregated for the two samples (instead of the standard three). To account for this uneven sampling, we included the number of leaf replicates collected per plant as a random effect in the mite and fungal abundance models, and the number of leaf replicates explained only a small amount of variation in our models (mite model variance = 1.599, fungi model variance = 0.058). Additionally, in nine instances, the petiole of the leaf sample was damaged such that extrafloral nectary abundance could not be assessed. In these instances, extrafloral nectary abundance was aggregated for the available samples, and we included the number of replicates collected per plant as a random effect in the predator abundance model, where extrafloral nectary abundance is used as a predictor. The number of replicates explained only a small amount of variance in the model (variance < 0.001).

We also used a piecewise structural equation model to assess if interactions between guilds were mediated by indirect effects from warming. However, we did not find that our structural equation model added any additional intuition to the interpretation of our data. Additionally, the structural equation framework limited our ability to incorporate and interpret interaction terms between guild‐level predictors and the warming treatment, limiting our capacity to assess warming's impact on the relationships between species. Because the structural equation model did not improve or differ qualitatively from our interpretations based on our mixed effects model, we do not present the results from this model here. However, methods and results for our structural equation model are available in Figure S1 and Appendix S1.

## 3 Results

### 3.1 What Are the Direct Effects of Warming?

**FIGURE 2:**
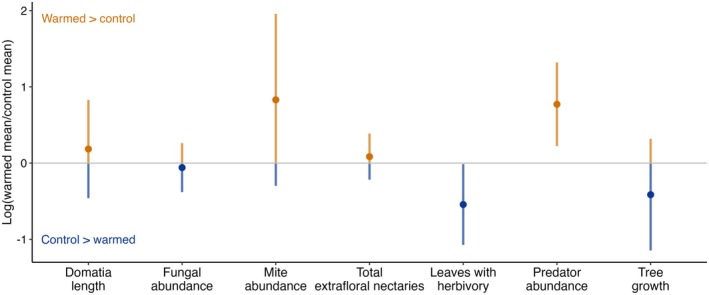
Effect sizes for each organism and trait measured in the experiment, as well as tree growth, with points showing the log response ratio of the means in the warmed treatment divided by the means in the control treatment for each variable. Error bars show the 95% confidence intervals.

On average, there were 116% more arthropod predators on seedlings in the warm plots compared to seedlings in the control plots (Figure 2). In addition, 42% more leaves had evidence of herbivory on seedlings in control plots compared to seedlings in warmed plots (Figure 2). Domatia length, fungal abundance, mite abundance, extrafloral nectary abundance, and tree growth otherwise did not differ significantly on seedlings in warmed versus control treatments (Figure 2).

Seedling mortality did not differ significantly between warming treatments (*z* = −0.298, *p* = 0.77).

### 3.2 Does Warming Alter the Relationships Among Multitrophic Interactors?

#### 3.2.1 Interactions Among Domatia, Mites, and Fungi

**FIGURE 3:**
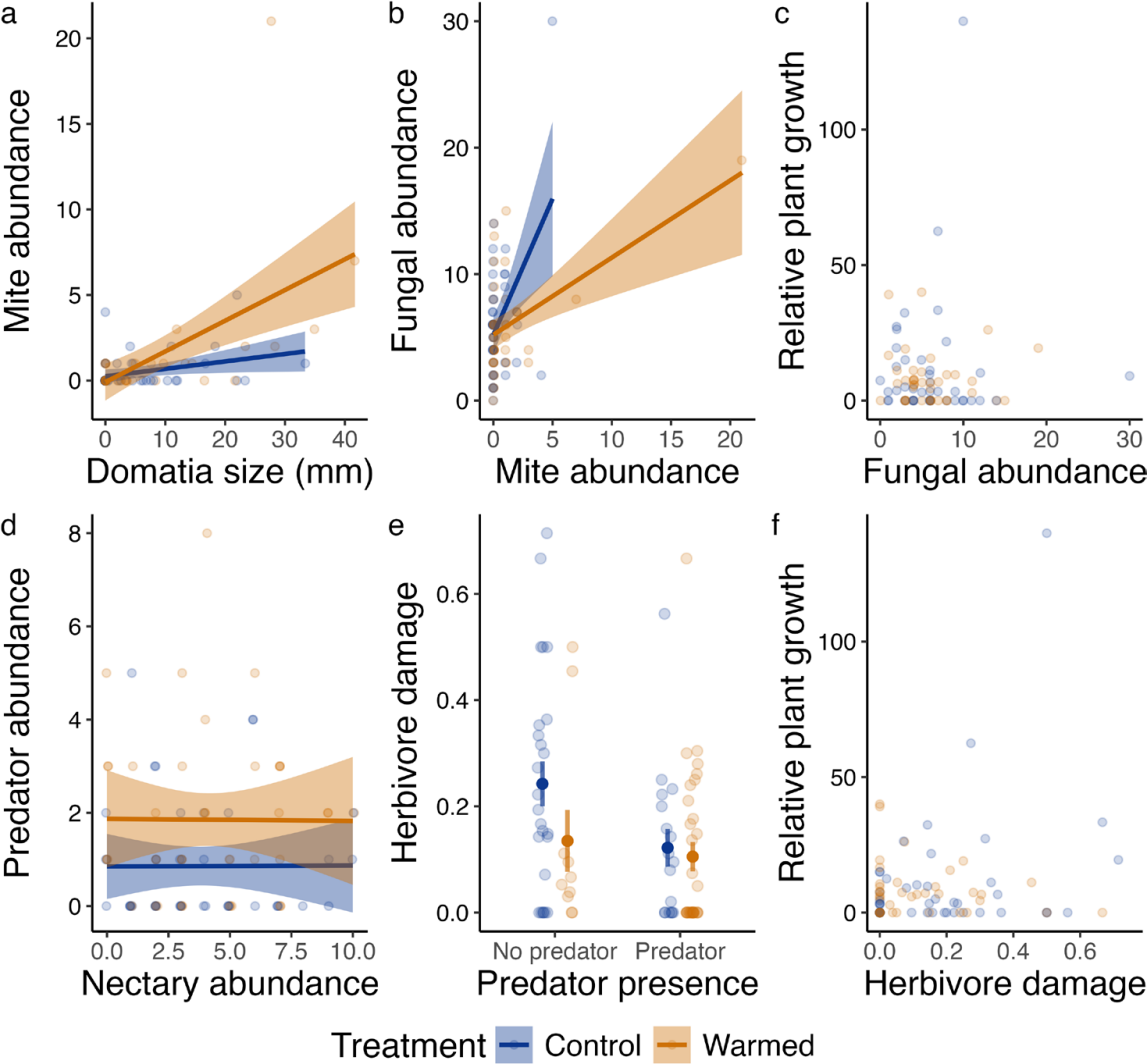
Visualizations of the relationships, as modified by the effects of warming, between trophic groups, including the relationship between (a) domatia size and mite abundance, (b) mite abundance and the abundance of leaf fungi (the number of hyphae along a 19 mm transect), (c) leaf fungi and plant growth, (d) extrafloral nectary abundance and arthropod predator (ants and spiders) abundance, (e) arthropod predator presence/absence and the proportion of leaves per plant exhibiting herbivore damage, and (e) herbivory and plant growth. Where a significant relationship is present, colored lines represent the lines best fit between each variable under warmed (orange) and control (blue) conditions, and shading indicates 95% CIs. In plot (e), the bolded points represent the mean, and bars represent the standard error. Transparent points in each plot represent the raw data.

Mite abundance was positively associated with domatia size (Table 2). While mite abundance was unaffected by warming (Table 2 and Figure 3a), there was a significant interaction between warming and domatia size (Table 2), with mite abundance increasing with domatia size more in warmed plots than in control plots (Figure 3a). The abundance of leaf fungi was positively associated with mite abundance (Table 2), but fungal abundance was unaffected by warming (*z* = −0.52, *p* = 0.60). Warming and mite abundance interacted significantly (Table 2), such that the relationship between fungal abundance and mite abundance was more strongly and positively associated in control plots than in warmed plots (Figure 3b). Neither fungal abundance (Table 2) nor warming (Table 2) affected plant growth, and there was no significant fungal abundance × warming interaction on plant growth.

**TABLE 2:** Summary statistics for the models describing mite abundance, fungal abundance, predator abundance, herbivore damage, and plant growth. In the test statistic column, the type of test statistic (*z* for generalized linear mixed effects models, *t* for linear mixed effects models) is noted. Where an interaction term was not significant, we removed it from the model and report model results without the interaction. Non‐significant interaction terms are demoted with a dash (−). Significant effects (*p* < 0.05) are bolded.

Response	Fixed effect	Estimate	SE	Test statistic	*p*
Mite abundance				*z*	
	Warming	−1.170	1.03	−1.14	0.26
	Domatia size	0.060	0.02	2.56	**0.01**
	Warming × domatia size	0.087	0.03	2.98	**< 0.01**
Fungal abundance				*z*	
	Warming	−0.069	0.13	−0.52	0.60
	Mite abundance	0.197	0.05	3.78	**< 0.01**
	Warming × mite abundance	−0.140	0.05	−2.69	**0.01**
Predator abundance				*z*	
	Warming	0.745	0.32	2.36	**0.02**
	EFN abundance	0.012	0.04	0.35	0.73
	Warming × EFN abundance	—	—	—	—
Herbivore damage				*t*	
	Warming	−0.082	0.06	−1.29	0.25
	Predator presence	−0.119	0.06	−1.97	**0.05**
	Warming × predator presence	—	—	—	—
Plant growth				*t*	
	Warming	−0.110	0.63	−0.18	0.87
	Fungal abundance	1.292	1.41	0.92	0.36
	Herbivore damage	−0.004	0.06	−0.07	0.94
	Warming × fungal abundance	—	—	—	—
	Warming × herbivore damage	—	—	—	—

#### 3.2.2 Interactions Among Extrafloral Nectaries, Arthropod Predators, and Herbivory

As described above (Figure 2), the abundance of predators was 116% higher in warmed plots than in control plots (Table 2 and Figure 3d), but predator abundance did not vary systematically with the number of extrafloral nectaries on leaves (Table 2), and there was no significant warming × extrafloral nectary interaction on the abundance of predators. The proportion of leaves showing evidence of herbivory was 39% lower on plants where at least one predator was present during the experiment (Table 2 and Figure 3e), but when considered in our generalized linear mixed effect modeling framework, there was no significant effect of warming on herbivory (Table 2 and Figure 3e), with no significant predator presence × warming interaction on herbivory. Neither herbivory (Table 2 and Figure 3f) nor warming (Table 2—as described above) affected plant growth, with no significant herbivory × warming interaction on plant growth.

## 4 Discussion

As climate change alters temperature regimes, warmer temperatures may alter the outcomes of tri‐trophic defensive interactions, yet the impact of warming may vary across different interactions and trophic groups, even on the same plant. Our results demonstrate that the impacts of warming differ among tri‐trophic interactions on black cherry. Specifically, we found that warming alters the relationships among black cherry, mites, and fungi, strengthening the positive relationship between mite abundance and domatia size under warming (Table 2), while weakening the positive relationship between mite abundance and fungal abundance (Table 2), even in the absence of direct effects on the abundances of the interacting species and traits (Figure 2). However, as further demonstrated through the tri‐trophic interaction between predators and herbivores, direct effects on the abundance of interacting species with warming need not necessarily result in altered relationships between species. Though warming increased the abundance of arthropod predators and decreased herbivory (Figure 2), these shifts did not translate to differences in the interactions between these groups (Table 2). Despite impacts on tri‐trophic interactions, there was no effect of temperature on plant growth, indicating plants may be robust to shifts in interactors with warming. Our findings further highlight that warming will not affect all species interactions uniformly, even when the nature of the interaction and the host plant species remain constant.

### 4.1 Domatia, Mites, and fungi

Higher temperatures increased the positive association between mites and domatia (Table 2 and Figure 3a) possibly indicating mites are using domatia more with warming. Mites are sensitive to desiccation risk (Grostal and O'Dowd 1994; Ghazy and Suzuki 2014), and one of the benefits of domatia to mites may be that they provide a refuge from abiotic stress, potentially by retaining moisture and higher humidity levels within domatia relative to the rest of the leaf surface (Pemberton and Turner 1989; Grostal and O'Dowd 1994; Romero and Benson 2005). To avoid potential desiccation from warmer conditions, mites could be using the protective services of domatia more under warmed conditions, making the availability of domatia more important, though warming did not appear to increase the size of domatia (Figure 1).

We also found a positive relationship between mite abundance and fungal abundance, a relationship that was weaker under higher temperatures (Table 2 and Figure 3b), counter to our expectations that mite abundance would decrease fungal abundance if there is top‐down control by mites (Norton et al. 2000). One possible explanation for this positive association between mite and fungal abundance is that we are instead seeing bottom‐up control by the fungi: leaves with more fungi can support more mites. Another non‐mutually exclusive explanation is that mites may have a role not only in consuming fungi on plant leaves but also in dispersing fungi to plant leaves. Predatory mites are used commercially to disperse fungal pathogens to their prey (Lin et al. 2017, 2019), and phoretic mites contribute to dispersing plant pathogens along with their hosts (Lombardero et al. 2003; Moser et al. 2010). The role of foliar mites in curating the leaf microbiome is still relatively understudied, though domatia‐dwelling mites are known to control pathogenic fungi in some well‐studied systems (Norton et al. 2000). Future work examining the mite and fungal community composition could disentangle the impacts of warming on specific taxa or functional groups, and mechanistic tests exploring the fungal microbiome with and without the presence of mites with warming would be valuable, particularly given that mite domatia are common across woody plants (Myers et al. 2024). Finally, we note that the significant interactions between domatia and warming on mite abundance, and between mite abundance and warming on fungal abundance, might be influenced by a handful of outliers (Figure 3a,b). While the significant interaction we detected is affected by the inclusion of these points, we have no reason to consider these values erroneous, and we therefore retain them in the analysis and reporting of our results.

### 4.2 Extrafloral Nectaries, Predators, and Herbivory

Warming increased the number of arthropod predators on plants (Figure 2), a result consistent with other studies (Prather et al. 2018; Høye et al. 2020; Magnoli et al. 2023; Hu et al. 2024). Contrary to our expectations, extrafloral nectary abundance did not increase predator abundance, even when we analyzed the abundance of ants and spiders separately (Table S1). It is possible that the extrafloral nectaries on our plants were producing low amounts of or no nectar during our study period, but we did not measure nectar production. Extrafloral nectary production in black cherry typically peaks in early spring, immediately following budburst (Tilman 1978), potentially indicating nectary production may not be a significant attractant to arthropod predators later in the growing season in this system. Regardless, extrafloral nectary abundance did not systematically vary in response to warming in our system, nor did it appear to mediate the responses of arthropod predators to warming (Table 2).

Warming also slightly reduced the amount of herbivory on plants (Figure 2), counter to expectation (O'Connor 2009; Hamann et al. 2021), as did arthropod predator presence (Table 2 and Figure 3e). Despite our finding that predator abundance increased with warming, and that herbivory decreased with predator presence, there was no significant interaction between warming and predator presence on the amount of herbivory plants experienced. One explanation for why an increase in the abundance of predators does not translate into an increase in top‐down control may be due to shifts in the composition of predator communities attending plants with warming. For example, warming may increase the abundance of less effective ant mutualist species (Barton and Ives 2014; Fitzpatrick et al. 2014), though these compositional shifts may not always result in differences in the mutualistic services ants provide (Stuble et al. 2013). Alternatively, higher temperatures may make predators less aggressive (Barton and Ives 2014, but see Zhou et al. 2017), which could reduce the impact these mutualists have on herbivory under warming. In this system, it could be that reduced aggression translates into no net change in interaction outcomes even as predator abundance increased. We did not identify or assess the behavior of the ants and spiders in this study, and as such, we are not able to assess if community composition or aggression differed between warming treatments. Future studies could further assess how compositional and behavioral shifts among interacting species influence the functioning of defensive mutualisms. As opposed to increased predator pressure, the reduction in herbivory we saw with warming may be due to other factors, including changes in plant palatability (Zhang et al. 2019), the expression of chemical defenses (Zvereva and Kozlov 2006; Bidart‐Bouzat and Imeh‐Nathaniel 2008), or food quality (Jamieson et al. 2015), pointing to potential additional avenues of study to explore relating to plant‐herbivore interactions and temperature shifts.

### 4.3 Impacts on Plant Performance

Notably, differences in the abundances of and relationships between interacting species did not translate into shifts in plant performance (Table 2). This finding indicates that plants may be robust to variation in species interactions with warming, counter to other studies that have explored the effects of multitrophic interactions and warming on plant performance (Marquis et al. 2014; Magnoli et al. 2023). Of course, maybe our metric for plant performance—growth—may not entirely capture the myriad impacts warming imposes on plants. Marquis et al. (2014) used plant stress (using variable fluorescence divided by maximum fluorescence) as their metric for plant performance, which they saw increase with warming due to differences in predator and enemy abundances. Plant stress, which we did not assess in our study, may not translate to changes in plant growth or other fitness metrics (Gotoh et al. 2018; Howard et al. 2020; Chavan et al. 2022). Additional studies relating multiple metrics of plant performance, including growth, stress, and other fitness metrics, to shifts in multitrophic interactions with warming would further improve our understanding of the effects plants incur from these shifts. Finally, it may be that plants are more responsive to certain interactions than others with warming; for example, a study exploring the effects of warming and mutualistic partner presence in annual forb *Chamaecrista fasciculata* found that warming increased the benefits plants gained from rhizobia, but the benefit plants received from ant mutualists was the same regardless of temperature conditions (Magnoli et al. 2023). It could be that interactions that mediate abiotic stress, such as rhizobia, are more important in shaping plant responses to warming, while interactions mediating biotic stressors may have similar outcomes regardless of abiotic conditions. Experimentally manipulating interacting species in this experiment would have increased our ability to identify mechanisms for the patterns described here and aided our ability to infer how each species or trophic group influenced plant growth. Additional studies factorially manipulating the presence of partner mutualists and/or enemies with warming would further improve our understanding of how each of these interacting species mediates plant responses to warming. Regardless, our findings suggest that, even with shifts in interactor abundances and relationships with warming, these effects do not necessarily cascade to influence plant performance.

An additional limitation of our study is that we only assessed differences in species abundances and interactions with warming for a single growing season. While this gives us insight into the short‐term effects of warming on our system, the impact of warming on interacting communities may vary with timescale. For example, warming can influence the phenology of species and alter temporal overlap among interacting organisms (Kharouba et al. 2018), which may reshape interactions through time. Similarly, the spatial scale of our study may also not fully encapsulate species' responses to warming. For example, simulated warming may be more impactful for less mobile trophic groups, such as mites, than for more mobile trophic groups, such as arthropod predators, which likely have a larger foraging range than is encompassed by our warming plots (Dornhaus and Powell 2009). Future research incorporating multi‐year data and observational studies that take advantage of natural temperature variation across a larger spatial scale would further refine our understanding of how warming reshapes multitrophic interactions beyond short‐term, small‐scale fluctuations (Magnoli et al. 2023).

## 5 Conclusions

Our findings indicate warming has the potential to shift tri‐trophic defense mutualisms, and that these shifts may not necessarily be tied to direct effects of temperature on the abundance of interacting species. In particular, we found that warming increased the positive association between mite abundance and domatia size by 8.7% (Table 2), while it weakened the positive relationship between mite abundance and the abundance of foliar fungi by 14%. However, warmer conditions did not directly affect the abundances of any of these groups (Figure 2). Further, while warming increased the abundance of arthropod predators by 116% and decreased the amount of herbivory plants experienced by 42%, it did not modify the effect predators had on herbivory. These findings indicate warming has the potential to shift some but not all species interactions, even within the same plant system. Additional studies further disentangling the effects of warming on community composition dynamics within and across trophic groups, manipulating the presence and absence of interacting groups, and exploring the temporal effects of warming on these dynamics would further refine our understanding of warming's effects on species interactions and plant performance.

## Author Contributions

**Emma Dawson‐Glass:** formal analysis (lead), visualization (lead), writing – original draft (lead), writing – review and editing (equal). **Nathan J. Sanders:** formal analysis (supporting), supervision (equal), visualization (supporting), writing – original draft (supporting), writing – review and editing (equal). **Marjorie G. Weber:** conceptualization (lead), formal analysis (supporting), funding acquisition (lead), methodology (lead), project administration (lead), resources (lead), supervision (equal), visualization (supporting), writing – original draft (supporting), writing – review and editing (equal).

## Conflicts of Interest

The authors declare no conflicts of interest.

## Supporting information


**Data S1:** ece372151‐sup‐0001‐supinfo.docx.

## Data Availability

Data is available from the Dryad Digital Repository at https://doi.org/10.5061/dryad.18931zd8v.
