# Supplementary material for: Temperature‐Dependent Shifts in Multiple Indirect Defensive Interactions on Black Cherry
Source: Ecol Evol. 2025 Sep 18;15(9):e72151. doi: 10.1002/ece3.72151 (PMC12445286; doi:10.1002/ece3.72151)
Supplement: Supplementary file 1 — Data S1: ece372151‐sup‐0001‐supinfo.docx. [file ECE3-15-e72151-s001.docx]

**Journal name:** Ecology and Evolution

**Manuscript type:** Research article

**Manuscript title:** Temperature-dependent shifts in multiple tri-trophic defense interactions on black cherry

**Authors:** Dawson-Glass, Emma^*,1^; Sanders, Nathan J.^1^; Weber, Marjorie G.^1^

^1^Department of Ecology and Evolutionary Biology, University of Michigan, Ann Arbor, MI 48109, USA

***Corresponding author:** Emma Dawson-Glass, Department of Ecology and Evolutionary Biology, University of Michigan, Ann Arbor, MI 48109, USA, email: [emmahdg@umich.edu](mailto:emmahdg@umich.edu)

**Appendix S1.** We built a piecewise structural equation model to assess the direct and indirect effects of warming on multitrophic interactions (psem function, piecewiseSEM package; Lefcheck, 2016) using a series of linear and generalized mixed effects models. We used the same models as described in our main manuscript to model herbivory, mite abundance, fungal abundance, and plant growth. For predators, we used presence/absence values representing whether a predator was ever present on the plant over the 10-week experiment period as our response variable (instead of total predator abundance, as in our main manuscript) because we use predator presence/absence as predictor variable in our herbivory model, and predator presence/absence was a better predictor for herbivory than total abundance. We included additional models for extrafloral nectar number (using a generalized linear mixed effects model with a poisson distribution) and domatia size (linear mixed effects model, square root transformed), both with treatment as a predictor variable. We included plot as a random effect for all models.

**References**

Lefcheck J.S. 2016. piecewiseSEM: Piecewise structural equation modeling in R for ecology, evolution, and systematics. *Methods in Ecology and Evolution*, 7:573-579.

**Figure S1.** Path diagram representing the results of our piecewise structural equation model representing the relationships between interacting species and warming. Black and grey lines represent significant and non-significant adjusted paths of the piecewise structural equation model, with marginal R^2^ values for endogenous variables shown. Model fit: Fisher’s C = 40.606, *p* = 0.094, d.f. = 25.

**
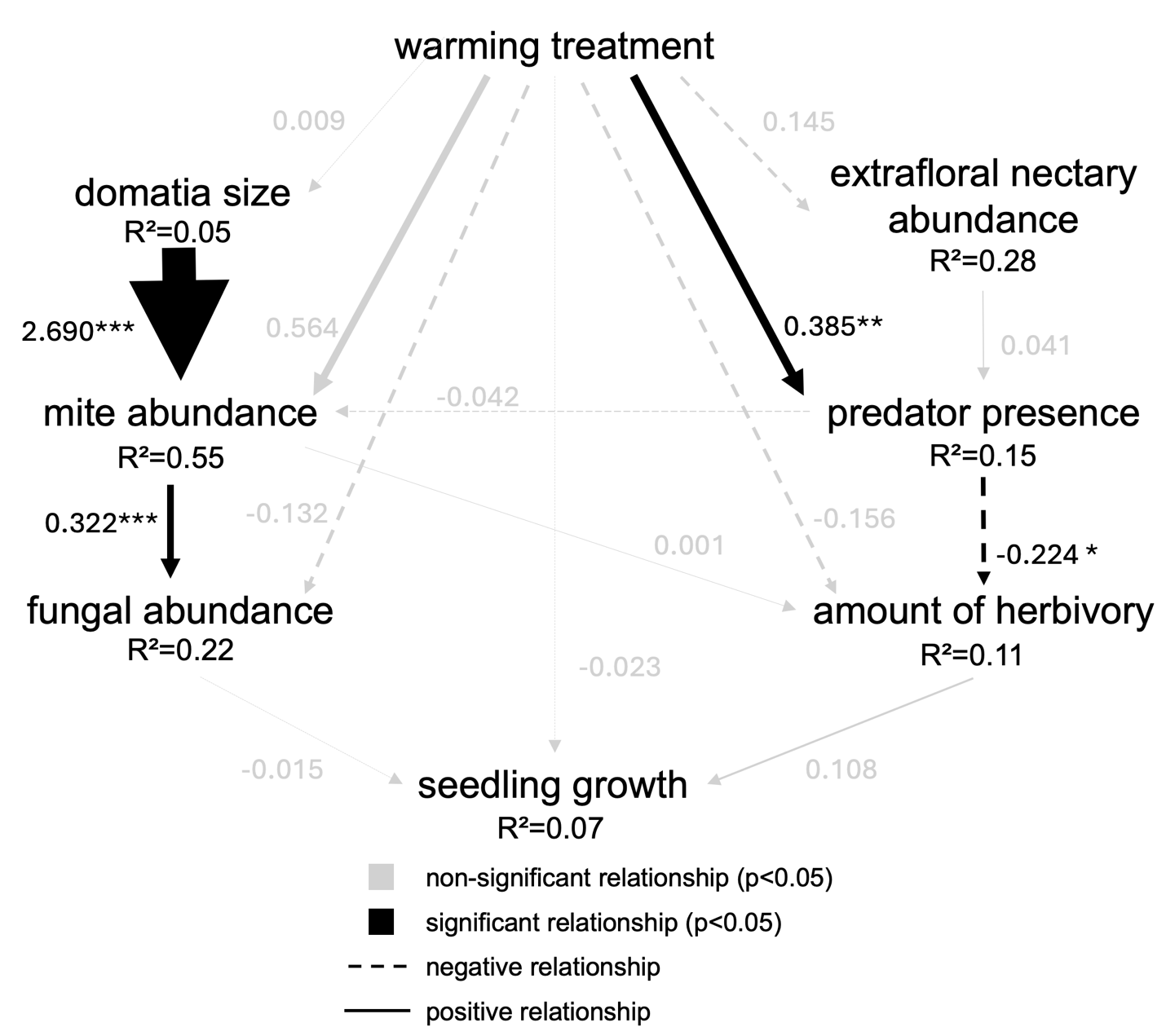
**

**Table S1.** Results generalized linear mixed effects model (fitted with a poisson distribution) of extrafloral nectary number and warming treatment on spider and ant abundances separately. Significant values (p < 0.05) are in bold.

|  | Warming | | |  | EFN number | | |
| --- | --- | --- | --- | --- | --- | --- | --- |
| Predator | Est. | *z* | *p* |  | Est. | *z* | *p* |
| Ants | 1.14 | 1.91 | 0.06 |  | 0.10 | 1.30 | 0.19 |
| Spiders | **0.68** | **2.35** | **0.02** |  | -0.01 | -0.38 | 0.71 |
